# *Bifidobacterium animalis* subsp. *lactis* BB-12 Protects against Antibiotic-Induced Functional and Compositional Changes in Human Fecal Microbiome

**DOI:** 10.3390/nu13082814

**Published:** 2021-08-17

**Authors:** Daniel Merenstein, Claire M. Fraser, Robert F. Roberts, Tian Liu, Silvia Grant-Beurmann, Tina P. Tan, Keisha Herbin Smith, Tom Cronin, Olivia A. Martin, Mary Ellen Sanders, Sean C. Lucan, Maureen A. Kane

**Affiliations:** 1 Department of Family Medicine, Georgetown University Medical Center, Washington, DC 20057, USA; tpt6@georgetown.edu (T.P.T.); kas224@georgetown.edu (K.H.S.); tcronin36@gmail.com (T.C.); 2 Department of Human Science, School of Nursing and Health Studies, Georgetown University Medical Center, Washington, DC 20057, USA; 3 Institute for Genomic Sciences, Departments of Medicine and Microbiology and Immunology, University of Maryland School of Medicine, Baltimore, MD 21201, USA; SBeurmann@som.umaryland.edu (S.G.-B.); OMartin@som.umaryland.edu (O.A.M.); 4 Department of Food Science, The Pennsylvania State University, University Park, PA 16802, USA; rfr3@psu.edu; 5 Department of Pharmaceutical Sciences, University of Maryland School of Pharmacy, Baltimore, MD 21201, USA; tian.liu@rx.umaryland.edu; 6 Department of Surgery, University of Maryland School of Medicine, Baltimore, MD 21201, USA; 7 Dairy & Food Culture Technologies, Centennial, CO 80122, USA; mes@mesanders.com; 8 Department of Family and Social Medicine, Albert Einstein College of Medicine, Montefiore Health System, Bronx, NY 10461, USA; slucan@yahoo.com

**Keywords:** abundance, antibiotic-induced perturbation, diversity, gut microbiota, probiotic, *Bifidobacterium*, short-chain fatty acid

## Abstract

The administration of broad-spectrum antibiotics is often associated with antibiotic-associated diarrhea (AAD), and impacts gastrointestinal tract homeostasis, as evidenced by the following: (a) an overall reduction in both the numbers and diversity of the gut microbiota, and (b) decreased short-chain fatty acid (SCFA) production. Evidence in humans that probiotics may enhance the recovery of microbiota populations after antibiotic treatment is equivocal, and few studies have addressed if probiotics improve the recovery of microbial metabolic function. Our aim was to determine if *Bifidobacterium animalis* subsp. *lactis* BB-12 (BB-12)-containing yogurt could protect against antibiotic-induced fecal SCFA and microbiota composition disruptions. We conducted a randomized, allocation-concealed, controlled trial of amoxicillin/clavulanate administration (days 1–7), in conjunction with either BB-12-containing or control yogurt (days 1–14). We measured the fecal levels of SCFAs and bacterial composition at baseline and days 7, 14, 21, and 30. Forty-two participants were randomly assigned to the BB-12 group, and 20 participants to the control group. Antibiotic treatment suppressed the fecal acetate levels in both the control and probiotic groups. Following the cessation of antibiotics, the fecal acetate levels in the probiotic group increased over the remainder of the study and returned to the baseline levels on day 30 (−1.6% baseline), whereas, in the control group, the acetate levels remained suppressed. Further, antibiotic treatment reduced the Shannon diversity of the gut microbiota, for all the study participants at day 7. The magnitude of this change was larger and more sustained in the control group compared to the probiotic group, which is consistent with the hypothesis that BB-12 enhanced microbiota recovery. There were no significant baseline clinical differences between the two groups. Concurrent administration of amoxicillin/clavulanate and BB-12 yogurt, to healthy subjects, was associated with a significantly smaller decrease in the fecal SCFA levels and a more stable taxonomic profile of the microbiota over time than the control group.

## 1. Introduction

The gut microbiota comprises hundreds of bacterial species and is an important factor in determining the health status of the host [1]. The key species of the gut microbiota ferment undigested carbohydrates that reach the colon, to produce short-chain fatty acids (SCFAs), lactic acid, H_2_, and CO_2_, as metabolites. SCFAs are essential for gastrointestinal health and are absorbed by colonic epithelial cells, and stimulate Na^+^-dependent fluid absorption [2], thereby conserving energy, Na^+^, and fluid. The administration of antibiotics impacts gastrointestinal tract homeostasis, and is associated with an overall reduction in the numbers and diversity of the gut microbiota, decreased SCFA production, accumulation of luminal carbohydrate, subsequent pH changes, decreased water absorption, and, potentially, antibiotic-associated diarrhea (AAD) [3,4,5,6,7,8,9,10]. However, the etiology of AAD is not fully elucidated.

AAD is more than a bothersome adverse event, it is associated with prescription noncompliance, morbidity, and overuse of second-line antibiotics. The rate of diarrhea associated with antibiotic usage is 5–25%, with 1–2% of those patients testing positive for *Clostridioides difficile*, which, in some cases, can result in serious illness [5,11,12,13,14,15,16,17,18,19,20].

Probiotics are live microorganisms that, when administered in adequate amounts, confer a health benefit on the host [21]. While there are robust clinical trials documenting the efficacy of some probiotics in preventing AAD, the mechanism(s) of how probiotics prevent AAD are unclear. However, restoration of metabolic function, including SCFA production by the microbiota, may be one means to mitigate AAD. Few studies have measured the impact of probiotics on the gut microbiota community structure and function, especially in the context of the amelioration of antibiotic-induced disturbances. Therefore, well-designed human studies measuring these endpoints, using well-characterized probiotics, are important to advance the mechanistic understanding of probiotic function and, ultimately, clinical use.

The probiotic *Bifidobacterium animalis* subsp. *lactis* BB-12 (BB-12) is a well-studied, well-characterized, and widely used probiotic. It produces acetate in vitro [22] and in vivo in preterm infants. Our aims in this study were to assess how a widely prescribed, broad-spectrum antibiotic impacts the fecal levels of SCFAs and the taxonomic composition of the gut microbiota, and to evaluate whether our chosen probiotic could ameliorate any of these changes. We hypothesized that concomitant administration of BB-12 would protect against a reduction in fecal acetate levels in subjects receiving antibiotics. If observed, such a protective function could contribute to understanding the mechanism of the probiotic-mediated prevention of AAD.

Our intervention, yogurt including BB-12, has been researched under an investigational new drug application (IND), as a live biotherapeutic product that is approved for study by the Food and Drug Administration (FDA) Center for Biologics Evaluation and Research (CBER). The genome sequence of BB-12 is published [23]. Further, BB-12 is the subject of a generally recognized as safe notice to the FDA, and has been evaluated in many human feeding and intervention trials [22], including four trials conducted by our group under this IND [24,25,26,27]. Few probiotic strains have been studied under an IND.

## 2. Materials and Methods

### 2.1. Study Design and Regulatory Approval

A mechanistic, randomized controlled study was conducted with two parallel arms, in a 2:1 fashion, with more participants in the BB-12 group than in the control group. The study protocol was approved by the Georgetown University Institutional Review Board and registered at ClinicalTrials.gov (NCT#03755765). An independent Data Safety Monitoring Board reviewed the protocol before study initiation and at time points throughout the study that were determined a priori. The study was conducted under IND No. 13691 FDA/CBER. 

**Figure 1:**
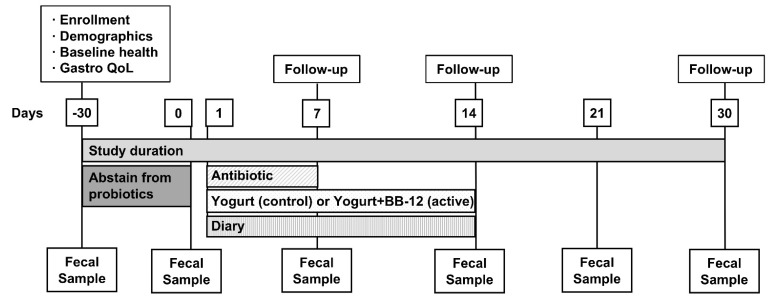
Participant timeline and sample/data collection schedule.

After the design and possible implications of this study were explained, written informed consent was obtained for each participant prior to any research activities. Following the completion of the informed consent and enrollment procedures, participants commenced a 30-day run-in period, during which they refrained from all probiotics. After completion of the 30-day run-in, participants began the antibiotic and yogurt interventions on day 1 (Figure 1). Each participant received a 7-day course of amoxicillin/clavulanate, 875 mg taken twice daily, and a 14-day supply of either a probiotic yogurt supplemented with BB-12, or a control yogurt. We selected this dose of amoxicillin/clavulanate because it is one that is often prescribed. Although there are other antibiotics that cause more AAD along with potential mucosal damage, we believed that it would be unethical to study such antibiotics in humans when they are not medically indicated. Participants were instructed to consume a 100 mL daily serving of the study yogurt concomitantly with the amoxicillin/clavulanate. Fecal samples used for SCFA and microbiome analyses were collected as shown in Figure 1.

### 2.2. Participants

Participants were generally healthy individuals between 18 and 65 years of age and were recruited from the community. Participants were required to be able to read, speak and write in English, and have a refrigerator and telephone access. Individuals were excluded for diabetes or asthma that required daily medication, allergy to strawberry, active diarrhea (defined as three or more loose stools per day for two consecutive days), any gastrointestinal medications, such as medicines for irritable bowel syndrome, gastroesophageal reflux disease, inflammatory bowel disease (a full medication list was reviewed by the principal investigator prior to enrollment), lactose intolerance, history of heart disease, including valvulopathies or cardiac surgery, any implantable device or prosthetic, history of gastrointestinal surgery or disease, milk-protein allergy, allergy to any component of the product or the yogurt vehicle, allergy to penicillin or cephalosporin class antibiotics, or actively breastfeeding, pregnant, or planning to become pregnant during the study. Participants agreed to refrain from all other probiotic foods and supplements throughout the study, starting from the 30-day run-in period through day 30 of starting the interventions.

### 2.3. Interventions

The control and BB-12 interventions were strawberry-flavored yogurt beverages developed and manufactured at the Pennsylvania State University. See Supporting Information Table S1 for the nutrient profile of the yogurt. The live culture used as the fermentation starter was YF-L702 (Chr. Hansens, Milwaukee, WI, USA), which contained *Streptococcus thermophilus* and *Lactobacillus bulgaricus*. Aside from the additional inoculation of the active product with BB-12, both yogurts were manufactured in the same manner. The microbiological composition of the active BB-12-supplemented yogurt at the end of its 30-day shelf life met targets of at least 1 × 10^10^ colony forming units per 100 mL serving of BB-12. Both interventions have been used in multiple studies and the manufacturing processes have been previously described in detail [25,27].

### 2.4. Blinding

The appearance, taste, nutritional composition (proteins, carbohydrates, lipids and energy) and packaging of the BB-12-supplemented and control products were identical, effectively blinding the participants. Six different bin numbers were assigned to the interventions, of which four corresponded to the BB-12 yogurt and two corresponded to the control yogurt. The principal investigator and research personnel at Georgetown University were blinded. All research personnel at the University of Maryland who conducted microbiome assessments and SCFA analysis were also blinded. Personnel at the Pennsylvania State University dairy plant who were involved with the production and labeling were not blinded; however, they did not have any participant contact, or involvement in data collection or analyses.

### 2.5. Randomization

Each participant was allocated in a truly concealed manner; research personnel had no methods to alter randomization or enrollment, nor had any knowledge of group assignment while participants were being followed. Participants were randomly allocated to either the BB-12 or control arm in a 2:1 ratio, using permuted block sizes of 6 and 9 in random order.

### 2.6. Compliance

We assessed adherence to the interventions in three distinct manners. First, we reviewed the daily assessment diaries. Second, participants sent photographs of their remaining antibiotics and yogurt bottles to research personnel at the end of the intervention periods. Third, at all follow-up phone calls, participants were asked about their antibiotic and yogurt intake on each day of the intervention period, and the amounts consumed. We defined protocol compliance as the consumption of 2 or more ounces of the assigned study yogurt per day, for at least 11 of the 14 days.

### 2.7. SCFA Analysis

Primary fecal SCFAs produced by anaerobic gut microbiota in the colon (acetate, propionate, butyrate) were quantified via a liquid chromatography–tandem mass spectrometry assay adapted, in part, from Han et al. [28]. Acetate was used as our primary measure of the effect of BB-12 on SCFAs; however, we also measured other primary SCFAs produced from gut microbiota, including propionate and butyrate. Stool samples used to assess SCFA levels were from the post run-in (day 0) and days 7, 14, 21, and 30. Authentic SCFAs, including acetic acid (acetate), propionic acid (propionate), and butyric acid (butyrate), were purchased from Sigma-Aldrich (St. Louis, MO, USA). SCFA internal standards (IS) including acetic acid-d4, propionic acid-d5, and butyric acid-d7 were also purchased from Sigma-Aldrich. Analytical reagent-grade 3-nitrophenylhydrazine (3NPH) HCl (97%), N-(3-dimethylaminopropyl)-N0-ethylcarbodiimide (EDC) HCl, and HPLC grade pyridine were purchased from Sigma–Aldrich. Liquid chromatography–mass spectrometry (LC–MS) grade acetonitrile, water, and formic acid were purchased from Fisher Scientific.

Feces from participants were stored at −80 °C until assayed. To prepare SCFA samples, feces were weighed and added to 50% aqueous acetonitrile to make a 20 mg/mL homogenate. Homogenates were vortex mixed for 5 min and then centrifuged at 4000× *g* at 10 °C for 10 min to extract SCFA. Then, 10 µL of the 20 mg/mL feces extract (supernatant) was combined with 10 µL of an IS working solution in 50% aqueous acetonitrile, 10 µL of 120 mM EDC/6% pyridine in 50% aqueous acetonitrile, and 10 µL of 200 mM 3-NPH in 50% aqueous acetonitrile. The mixture was incubated for 15 min at 40 °C and was then diluted to 1 mL with acetonitrile. The IS working solution was 2 mM acetic acid-d4, 100 µM propionic acid-d5, and 100 µM butyric acid-d7 in 50% aqueous acetonitrile.

Liquid chromatography–tandem mass spectrometry (LC-MS/MS) was performed using a Dionex Ultimate 3000 RS liquid chromatography system coupled to a TSQ Altis tandem quadrupole mass spectrometer equipped with a heated electrospray ionization source operated in negative ion mode. Separation of SCFA was effected on a Phenomenex Kinetex C18 column (100 Å, 2.6 µm, 100 mm × 2.1 mm) using eluent A as water with 0.01% formic acid and eluent B as acetonitrile with 0.01% formic acid. The following linear gradient separation was used: (time, % B)−1.5 min, 20% B; 0 min, 20% B; 2 min, 25% B; 3 min, 25% B; 3.1 min, 80% B; 4 min, 80% B. The flow rate was 0.5 mL/min, the column temperature was 30 °C, and the injection volume was 1 µL. Mass spectrometry parameters were as follows: spray voltage, 2500 V; sheath gas, 50 arbitrary units (a.u.); auxiliary gas, 10 a.u.; sweep gas, 1 a.u.; ion transfer tube temperature, 350 °C; vaporizer temperature, 300 °C; cycle time, 0.4 s; Q1 resolution, 0.7 FWHM; Q3 resolution, 0.7 FWHM; CID gas, 2 mTorr. SCFA were detected according to a unique selected reaction monitoring (SRM) mass transition as the SCFA-3-NPH derivative. Acetic acid, propionic acid, and butyric acid derivatives all had a quantitation *m*/*z* transition and a qualifier *m*/*z* transition for confirmation. Each IS derivative had one *m*/*z* transition. The *m*/*z* transition, retention time, collision energy, and RF lens values for each analyte were as follows: acetic acid-3-NPH (quant), 194→152, 1.23 min, 14 V, 58 V; acetic acid-3-NPH (qual), 194→137, 1.23 min, 18 V, 58 V; acetic acid-3-NPH-d4, 198→154, 1.22 min, 14 V, 58 V; propionic acid-3-NPH (quant), 208→165, 1.82 min, 12 V, 58 V; propionic acid-3-NPH (qual), 208→137, 1.82 min, 18 V, 58 V; propionic acid-3-NPH-d5, 227→138, 1.79 min, 14 V, 58 V; butyric acid-3-NPH (quant), 222→152, 2.82 min, 15 V, 63 V; butyric acid-3-NPH (qual), 222→137, 2.82 min, 19 V, 63 V; butyric acid-3-NPH-d7, 229→137, 2.77 min, 20 V, 68 V.

### 2.8. Microbial DNA Extraction and 16S rRNA Gene Sequencing

Genomic DNA was extracted from stool samples with the MagAttract microbial DNA kit (Qiagen) using a custom automated protocol on the Hamilton Microlab Star. Samples were thawed on ice and a 200 μL aliquot from the stool sample was used as input for the kit following the manufacturer’s protocol. Cells were lysed by bead beating on the TissueLyser (Qiagen) at 20 Hz for 20 min and the final elution volume was 110 μL. The V3–V4 regions of the 16S rRNA gene were amplified by two-step PCR, with amplicon pooling, sequencing on an Illumina HiSeq 2500 instrument, and sequence data processing as previously described [29].

Sequence reads from the 16S rRNA gene profiling are available on NCBI Sequence Read Archive under accession number PRJNA668752.

### 2.9. 16S rRNA Gene Sequence Analysis

Amplicon sequence variants (ASVs) generated by DADA2 were taxonomically classified using the RDP Naive Bayesian Classifier [30] trained with the SILVA v128 16S rRNA gene database [31] as implemented in the dada2 R package [32]. Amplicon sequence variants (ASVs) of major stool taxa were assigned species-level taxonomy using speciateIT (http://ravel-lab.org/speciateit (accessed on 16 July 2020)). The phyloseq R package [33] was used for analysis of the microbial community data. Shannon diversity was compared between control and BB-12 groups at each of the time points, as well as longitudinally, using repeated measures ANOVA. Pairwise comparisons were performed with post hoc Tukey HSD test.

Microbiota changes focusing on relative abundances of bacterial taxa were compared using linear discriminant analysis effect size (LEfSe) [34]. LEfSe determines the features (organisms, clades, OTUs, genes, or functions) most likely to explain differences between classes by coupling standard tests for statistical significance with additional tests encoding biological consistency and effect relevance. The Galaxy implementation of LEfSe (http://huttenhower.org/galaxy (accessed on 29 July 2020)) with default options was used [34]. Differences were evaluated via Kruskal–Wallis and Wilcoxon rank-sum testing, with α-value for the factorial Kruskal–Wallis test among classes and pairwise Wilcoxon test between subclasses of 0.05, and threshold for the logarithmic linear discriminant analysis score for discriminate features of 2.0.

In addition, Bray–Curtis dissimilarity was calculated to assess the beta diversity, which is a measure of how different the bacterial composition is in one sample compared to another (i.e., how different the taxonomic abundance profiles are among different samples). The differences in bacterial abundances between two samples are expressed in values ranging from 0 to 1, with 0 meaning that both samples share the same species at exactly the same abundances and 1 meaning that both samples have completely different species and abundances.

### 2.10. Sample Size Calculation

Sample size calculations were informed by Hoverstad et al. [35,36], who compared changes in the mean SCFA levels between baseline fecal samples and fecal samples collected from healthy volunteers after six days of administration of various antibiotics or placebo. In these studies, a mean reduction in acetate levels from 45 ± 16 μmol/g at baseline to 24 ± 18 μmol/g after antibiotic treatment (46% reduction in acetate) was observed. Reductions in acetate after antibiotic treatment with various antibiotics ranged from 5% to 79%. Ampicillin, a derivative similar to amoxicillin, was associated with a 38% decrease in acetate after antibiotic treatment [35,36]. We anticipated that we would see a similar decrease in acetate in our proposed study, assuming at least a 42% decrease in acetate in the antibiotic-treated group and no greater than a 15% decrease in the BB-12-treated group. Based upon this literature and using a ratio of volunteers in a BB-12-supplemented group and control group of 2:1, a total sample size of 60 subjects was calculated to be needed to have 80% power to detect a significant difference (*p* < 0.05) in mean acetate levels as a measure of gut microbiota production of SCFA between a control and antibiotic-treated group.

### 2.11. Other Data Collected

We also collected clinical data, including demographics, common clinical outcomes, adverse events, and dietary recall diaries. Diet was assessed via the healthy eating index–2015 (HEI-2015). The HEI-2015 includes 9 adequacy components (total fruit, whole fruit, total vegetables, greens and beans, whole grains, dairy, total protein foods, seafood and plant protein, and fatty acids) and 4 moderation components (refined grains, sodium, percentage of energy from added sugars, and percentage of energy from saturated fatty acids). The HEI-2015 scores each of the components on a density basis out of 1000 calories, with the exception of ratio of unsaturated to saturated fatty acids. The total HEI-2015 score ranges from 0 (nonadherence) to 100 (perfect adherence) [37,38]. Clinical data were examined using descriptive statistics.

## 3. Results

### 3.1. Participant Flow and Baseline Characteristics

**Figure 2:**
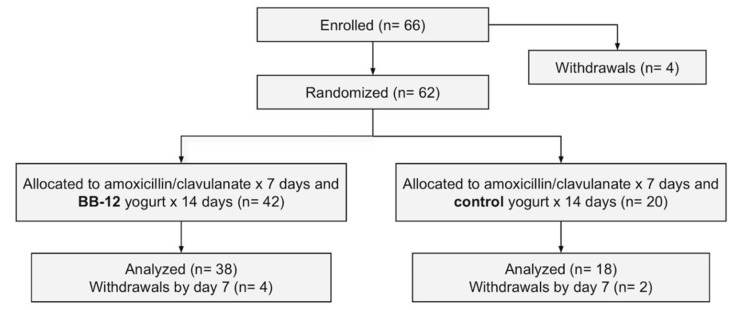
Consolidated standards of reporting trials (CONSORT) flow diagram of participants. Sixty-six participants were enrolled, 62 of which were randomized (42 to BB-12 group and 20 to control) after a 30-day run-in period. By day 7 of the intervention, 56 participants (38 BB-12 and 18 control) remained in the study.

The study participants were enrolled from July 2019 through to January 2020. During this time, 66 participants were initially enrolled, 62 of which were randomized after a 30-day run-in period. Forty-two participants were assigned to the BB-12 group and 20 participants to the control group. By day 7 of the intervention, 56 participants (38 BB-12 and 18 control) remained in the study (Figure 2, Table 1).

There were no significant baseline clinical differences between the two groups (Table 1). The average age was 29.4 years in the active group and 29.6 years in the control group. Most participants were single, had insurance, and did not use tobacco. Based on the dietary assessments that were conducted during the run-in period, the diets of both the groups were very similar, with average healthy eating index (HEI) scores of 61 in the active group compared to 58 in the control group. However, prior to the run-in period, 50% of the control group participants reported eating yogurt regularly, versus 68% of the active group participants. The probiotic content of the yogurts that were consumed is not known, but in the United States, the majority of yogurt that is sold contains live starter cultures and many also contain added probiotics. The baseline fecal SCFA levels were similar for acetate, propionate, and butyrate (data not shown) between the two groups.

### 3.2. Primary Outcomes after Antibiotic Administration Measured by SFCA Analyses

**Figure 3:**
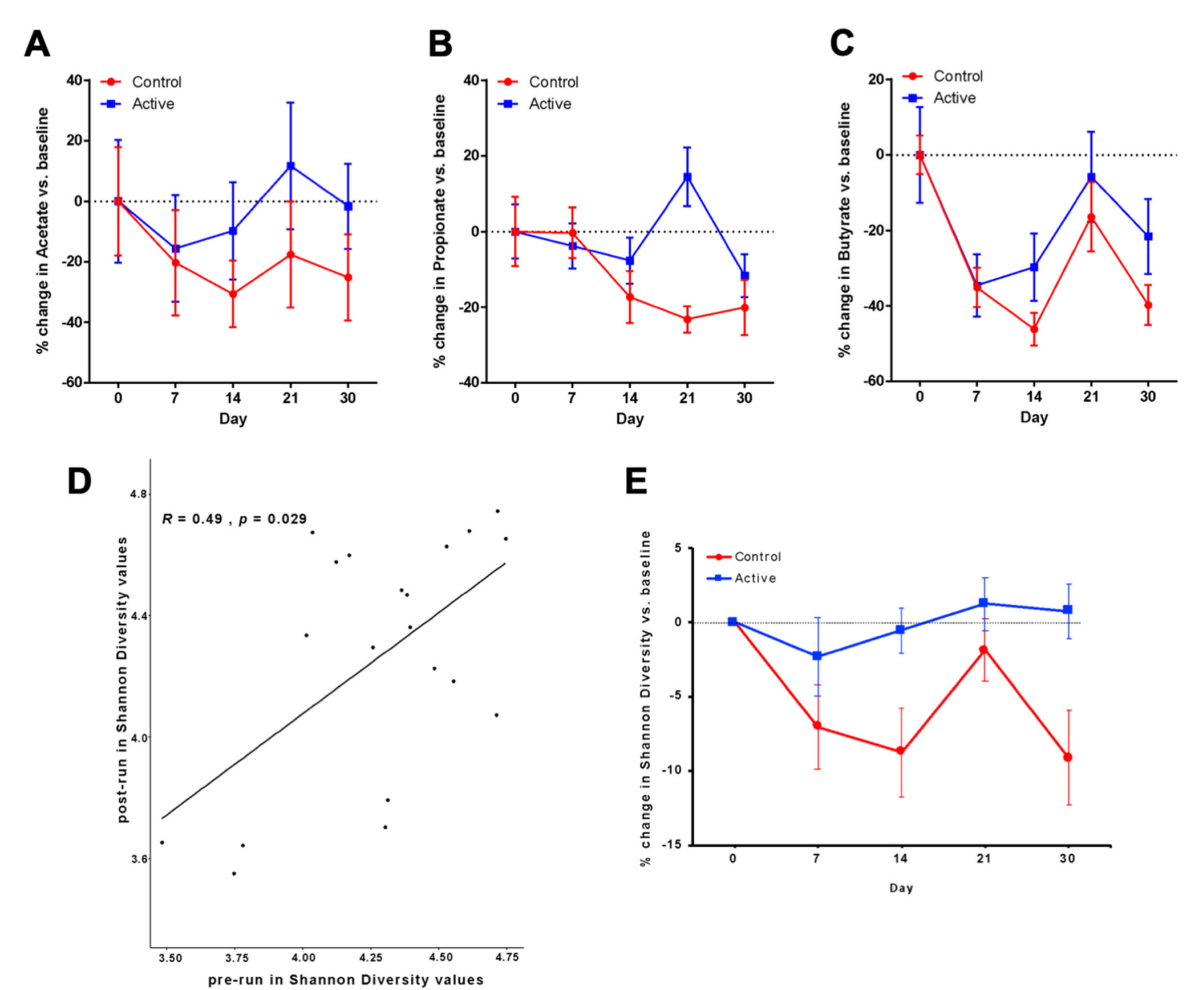
Comparisons of fecal SCFA levels and Shannon diversity at baseline with multiple time points post amoxicillin/clavulanate administration. (**A**–**C**) % change in average SCFA levels as compared to post run-in (day 0) baseline. (**A**) Acetate, (**B**) propionate, (**C**) butyrate. The acetate control group (red) in (**A**) remains decreased at day 30 (−25.1%) whereas active (blue, BB-12) group returns to baseline at day 30 (−1.6%). (**D**) Correlation of Shannon diversity between pre- and post-baseline values (Spearman’s rank correlation coefficient *R* = 0.49, *p* < 0.05). (**E**) Differences in Shannon diversity between the control (red) and BB-12 (blue) groups at time points pre (baseline 1, day 30), post (baseline 2, day 0), day 7, day 14, day 21, and day 30.

Our primary hypothesis was that antibiotics would cause a reduction in fecal SCFAs and that BB-12 supplementation would protect against antibiotic-induced SCFA reduction and/or be associated with a more rapid return to baseline SCFA levels, as compared to the control participants, who did not receive BB-12. Acetate was used as the primary measure of the effect of BB-12 on SCFAs, determined a priori. We quantified fecal acetate over time and calculated the percent change versus the post run-in baseline for each group (Figure 3A, Table 2). As hypothesized, we observed a significant decrease in the fecal acetate levels in both the control and BB-12 groups, following antibiotic administration (Figure 3A). The average percent decrease in acetate levels, compared to the baseline, ranged from 30.6% to 17.6%, with a decrease of 20.3% on day 7 and 25.1% on day 30. BB-12 administration was associated with significantly smaller antibiotic-induced decreases in fecal acetate levels than those observed in the control group. In the BB-12 group, the average decrease in acetate levels, after antibiotic treatment, ranged from 15.6% to 11.7%, with a change of −15.6% on day 7 and −1.6% on day 30. The BB-12 group also had a more rapid return of acetate levels to the baseline than the control group (Figure 3A). At day 30, the acetate levels for the control group remained decreased (−25.1%), whereas the acetate levels for the BB-12 group had returned to the baseline levels (−1.6%). Using the percent change versus baseline, we calculated a Cohen’s d of 0.6 at day 30, indicating a medium effect size for the BB-12 treatment effect.

### 3.3. Relative Risk for Improvement

We calculated the relative risk for improvement, as defined as the percentage of participants that improved in the BB-12 group versus the percentage of participants that improved in the control group at day 30. Improvement was defined as having a fecal acetate level within 15% of the post run-in baseline value. At day 30, the calculated relative risk for improvement of 2.3 indicates that the BB-12 treatment was 2.3-fold more likely to provide a benefit as compared to the control.

### 3.4. Area under the Curve Analysis of Change in Acetate

An area under the curve (AUC) analysis of the average percent change in acetate, from the baseline, was calculated (from Figure 3A). The control group had an AUC of −610 and the BB-12 group had an AUC of −90. The AUC analysis returns a negative area value, since the acetate levels decrease upon antibiotic treatment, which is reflected in a negative deviation from the baseline. This AUC analysis indicates that the control group has a 6.8-fold greater area, indicating a much greater deviation from the baseline than the BB-12-treated group.

### 3.5. Propionate and Butyrate Measured after Antibiotic Administration

In addition to acetate, we also quantified fecal propionate and butyrate at all the study time points (Figure 3B,C, Table 2). Butyrate and propionate showed attenuated antibiotic-induced decreases and more rapid return to the baseline in subjects receiving the BB-12 probiotic yogurt. The AUC analysis for butyrate and propionate yielded a negative area value, since the butyrate and propionate levels decrease from the baseline upon antibiotic treatment. The AUC for propionate in the control group was −399, whereas the AUC for propionate in the BB-12 group was −17. This reflects a 23.5-fold greater area for the control group, indicating that the control group had a greater deviation from the baseline as compared to the BB-12-treated group. The AUC for butyrate in the control group was −879 and the AUC for the BB-12 group was −594, which indicates that the control group had a 1.5-fold greater area and greater deviation from the baseline than the BB-12-treated group. The individual SCFA expressed as a percent of total SCFA were present in the expected ratios (Figure S3).

### 3.6. Microbiota Analyses

Gut microbiota analyses were carried out using the same fecal samples that were analyzed for SCFAs. The Shannon diversity values from the two baseline samples in all the participants were similar, with a mean of 3.76 (Figure 3D), and linear discriminant analysis effect size (LEfSe) analysis confirmed that the taxonomic composition of the two baseline samples in each treatment group were similar (Supporting Information Figures S1 and S2).

We observed a decrease in the Shannon diversity in the majority of the samples that were collected on days 7 and day 14, in both the control and BB-12 groups; however, the decrease in Shannon diversity was significantly greater in the control group at both the time points (Figure 3E). The differences in the Shannon diversity between the control and BB-12 groups persisted beyond the 14-day period of probiotic administration. At days 21 and 30, we continued to observe a greater decrease in community diversity in the control versus the BB-12 group.

### 3.7. Taxonomic Characteristics and Changes over Time

**Figure 4:**
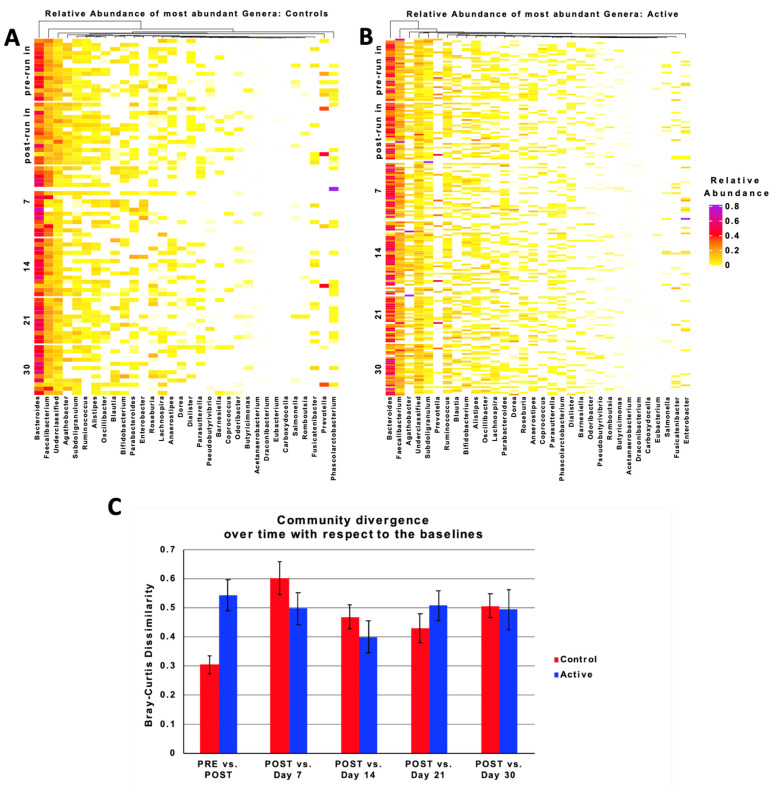
Taxonomic shifts induced by amoxicillin/clavulanate administration with and without BB-12 over time. (**A**,**B**) Hierarchical dendrogram of participants and their predominant fecal bacterial genera; control group (**A**) and BB-12 (active) group (**B**). The heat map represents the relative abundance (range 0–1; 1 = 100%) of each bacterial genus. The predominant genera are represented along the right *x*-axis. The legend for the heat map is provided on the right side representing the relative abundance of each bacterial genus within each sample. (**C**) Percent community divergence (Bray–Curtis dissimilarity) over time with respect to the baseline (post run-in, day 0). Control group (blue bar graphs) exhibits overall greater increase in community dissimilarity when compared to BB-12 recipients (red bar graphs).

To further explore the microbiota changes that are induced by amoxicillin/clavulanate, with and without BB-12, we characterized the taxonomic shifts over time in our samples. As shown in Figure 4A,B, *Bacteroides*, *Faecalibacterium*, and a collection of under-classified amplicon sequence variants (ASVs), represented the dominant taxa in the baseline samples that were collected from both the control and BB-12 subjects, along with a number of lower abundance members of the Firmicutes (including the genera *Agathobacter, Subdoligranulum, Ruminococcus, Blautia, Oscillibacter, Lachnospira*, and *Roseburia*) and Actinobacteria (including the genus *Bifidobacterium*) phylum. The administration of antibiotics in the absence of BB-12 was associated with a decrease in the relative abundance of 48 taxa, including several members of the Firmicutes phylum (*Streptococcus thermophilus*, *Streptococcus oralis*, *Eubacterium siraeum*, *Dialister propioifaciens*, and *Butyricoccus pullicaecorum)*, and an enrichment in *Bacteroides* and *Enterobacter*. This change was most pronounced at day 7, although the reductions in the relative abundance of several taxa persisted throughout the time course of this study. Similar taxonomic changes in the gut microbiota were observed at day 7 in the subjects receiving antibiotic plus BB-12; however, fewer taxa overall were affected, resulting in a more stable taxonomic profile over time, as revealed by the Bray–Curtis metrics (Figure 4C). Despite the antibiotic-associated changes in a number of bacterial taxa, the calculated Bacteroidetes:Firmicutes ratio was similar in both the BB-12 and control groups, at each time point in the study.

### 3.8. Relative Abundance

A comparison of the 16S rRNA gene amplicon datasets between the control and BB-12 groups demonstrated a difference in the relative abundance of *Bifidobacterium animalis* (of which BB-12 is a member) over the 14 day period that BB-12 was administered, as would be expected. There were no 16S rRNA gene sequences derived from *B. animalis* in any of the samples collected from the control group or BB-12 group at the baseline, and no BB-12 sequences in the day 7 or day 14 samples from the control group. By contrast, we did identify *B. animalis* sequences in the samples collected from the BB-12 group at day 7 and day 14. While the overall abundance of *B. animalis* was low, at 0.1% and 0.3% of the total sequences on days 7 and 14, respectively, these differences were significantly different than the baseline values. The identification of *B. animalis* in the samples collected on days 7 and 14, are consistent with the reports from subjects with regard to compliance. The absence of *B. animalis* in the BB-12 group, on days 21 and 30, suggests that this probiotic does not colonize the gut.

### 3.9. Clinical Outcomes and Adverse Events

The common clinical outcomes that were reported at each time point are listed in Table 3. By day 7, 42% of the control participants had at least one day of loose stools compared to 26% in the BB-12 group. All the adverse events were self-limiting, and there were no serious adverse events reported. A total of 53 adverse events were reported by 20 participants in the control group, compared to 66 total adverse events reported by 42 participants in the BB-12 group.

## 4. Discussion

Acetate is the most abundant SCFA, produced through anaerobic microbial fermentation in the human colon, and its production is reduced by antibiotic treatment [6,35,36]. Since BB-12 produces acetate in vitro, we hypothesized that acetate production by BB-12 may mediate, at least in part, the ability of this strain to reduce AAD. We therefore used fecal acetate levels as our primary outcome measure in this trial. Consistent with our hypothesis, the BB-12-containing yogurt attenuated the decrease in acetate after antibiotic treatment and produced a more rapid return to the baseline SCFA levels than the control yogurt without BB-12.

We observed a 25% average decrease in fecal acetate at day 30 in the control group, while the levels of acetate in the BB-12 group had returned to near the baseline levels at the same time points. On day 30, we also observed that the levels of propionate and butyrate were still greatly reduced in the control subjects, with butyrate being nearly 40% reduced from the baseline. These findings are consistent with previously published reports on the delayed and incomplete recovery of the gut microbiota in healthy subjects receiving antibiotics, as assessed by 16S rRNA gene profiling [4], and suggest that antibiotic perturbation of the gut microbiota may have long-term functional consequences.

The data from this clinical study are also consistent with our hypothesis that BB-12 supplementation can mitigate antibiotic-induced shifts in the microbiota (as evidenced by the results at all the time points in our study), and are associated with a quicker return to the baseline microbiota composition, as compared to the control group (as evidenced by the Bray–Curtis results at days 14, 21, and 30). We observed a larger difference in the Bray–Curtis metric between the pre- and post-run-in samples in the BB-12 group as compared to the control group. The reasons for this are not entirely clear; however, it is possible that this reflects the fact that participants were asked to refrain from the consumption of probiotics during the run-in period. The dietary recall data that were collected as part of this study revealed that participants in the BB-12 group were consuming more than twice the daily amount of probiotic foods and supplements than the control group, at the start of the run-in period. Therefore, the requirement to discontinue all probiotics during the run-in period was potentially of greater impact to the participants who were enrolled in the BB-12 group.

It is worth noting that a recent non-randomized study from Suez et al. [39] examined the effect of a product containing 11 strains of lactobacilli and bifidobacteria on the taxonomic composition and transcriptomics profiles of the gut microbiota, in participants given an antibiotic for 7 days, as we had conducted in our study. The study by Suez et al. [39] demonstrated that antibiotic treatment perturbed the gut mucosa microbiota populations and gut microbiota gene expression profiles, and that administration of the probiotic delayed the recovery of the perturbed microbiota. By contrast, our results showed that the probiotic BB-12 mitigates against the antibiotic disruption of gut microbial function, as assessed in fecal SCFA levels. Although these studies reached seemingly disparate results, there were several experimental differences between these two studies, including the administration of different antibiotics, different probiotic formulations, and different probiotic dosing schedules. Also relevant to the different outcomes in these two studies was the identification, by Suez et al. [39], of a secreted factor from one or more of the *Lactobacillus* species, in their multi-strain probiotic product, which inhibited the growth of the human microbiome in a host-free culture system in vitro. Such an inhibitory substance may have caused a delayed recovery of the antibiotic-challenged microbiome.

Given that the mechanisms of action among probiotic strains can differ substantially, this inhibitory factor could be unique to the preparation used by Suez et al. [39], and cannot be assumed to be expressed by other probiotic strains; caution must be taken when generalizing from a specific probiotic preparation to probiotics as a class. Therefore, it is not surprising that BB-12 could function in a manner that protects the microbiome, whereas the preparation used by Suez et al. [39] did not. Our intervention comprised a well-documented single probiotic strain, while Suez et al. [39] studied a commercially available product that they claimed to be a probiotic, yet no evidence of human intervention studies documenting any health benefit were referenced. We believe it prudent to avoid using the generic term, probiotic, whenever possible, and to only refer to microbial interventions as probiotics if they are live microorganisms that, when administered in adequate amounts, confer a health benefit on the host.

An important reason why our study may have demonstrated positive results may be the timing of the probiotic administration. As previously mentioned, a variety of probiotic strains have been demonstrated to help prevent both AAD and *C. difficile* infection (CDI), with one study showing that administration of probiotics closer to the first dose of antibiotic reduces the risk of CDI by >50% [40,41,42]. However, probiotics have not been shown to help treat CDI. In our study, the probiotic was started on the same day as the antibiotic, in contrast to Suez et al. [39], where the probiotics were not started until 7 days after starting the antibiotics. The potential importance of the timing of probiotic administration was also demonstrated in a clinical trial of *Lactocaseibacillus rhamnosus GG*, which found no effect in reducing the duration of acute pediatric gastroenteritis [43]. Previous studies (systematically reviewed by Szajewska et al. [44]), which had administered the probiotic soon after symptoms developed, demonstrated a benefit of *L. rhamnosus* GG for this endpoint. The null trial [43] recruited subjects up to 3 days (median of 53 h) after the onset of symptoms, a point at which spontaneous remission likely commenced. Although preferred timing of probiotic consumption has not been clearly elucidated, starting the probiotic as early as possible, before the disease has progressed, may result in greater opportunity for the probiotic mechanisms to be expressed and may ultimately lead to more beneficial clinical outcomes.

Certain probiotics are recommended for the prevention of AAD [45], but the mechanism(s) driving this clinical effect are not known. Data from several studies are consistent with the notion that the antibiotic-induced disruption of commensal bacteria in the colon results in a significant reduction in SCFA production (5–90% depending on antibiotic) and a concomitant reduction in Na^+^-dependent fluid absorption that results in AAD [6,35,36]. However, Clausen et al. reported that AAD was consistently related to reduced fecal SCFA concentrations and production rates, and that patients without altered bacterial metabolism (i.e., unaltered SCFA levels) did not experience AAD [6]. Taken together, our results support the idea that BB-12 attenuates AAD through sustaining acetate production and Na^+^-dependent fluid absorption.

Acetate produced by BB-12 could contribute to the maintenance of gut homeostasis, by the cross-feeding of certain commensals, such as members of the *Clostridium*, *Eubacterium,* and *Roseburia* genera, which use acetate to produce butyrate. Maintenance of SCFA levels in the colon have also been proposed to support gut health by reducing luminal pH, thereby inhibiting pathogens [46,47]. In addition, SCFAs have health-promoting effects directly on the host [48,49], although we did not explore this possibility in the clinical trial. As an additional application of our research, fecal acetate levels may be a useful metric in assessing the efficacy of probiotics to mitigate AAD.

Our results are consistent with our secondary hypothesis that BB-12 supplementation protects against antibiotic-induced shifts in the gut microbiota. In the control group, we saw a significant reduction in the relative abundance of several known butyrate producers from the Lachnospiraceae and Ruminococcaceae families at day 7, and these changes persisted through to day 30 (Figures S1 and S2). By contrast, while we observed a reduction in the relative abundance of many of these same taxa in the BB-12 group on day 7, these changes began to resolve at day 21, and by day 30 there were no differences observed in the relative abundance of a number of putative butyrate producers as compared to the baseline.

The acetate-producing ability of BB-12 likely drives our observations, and, as such, our results should be considered to be specific to this strain. However, members of the genus *Bifidobacterium* share a central metabolic pathway, known as the *Bifidobacterium* shunt, which yields lactate and acetate [50]. Therefore, acetate production is a shared function among *Bifidobacterium* probiotics [51]. However, the ability to produce acetate may not be sufficient to recapitulate BB-12’s functionality, as other strain-specific characteristics, such as the growth kinetics in situ, may also contribute to the observed outcomes.

This study was not powered to detect clinical changes, and almost no AAD was observed. However, 44% of the participants in the control group reported some loose stool at day 7, compared to 26% of the participants in the BB-12 group. Additionally, 44% of the control group reported some flatulence by day 7, while only 10% of the BB-12 group reported flatulence. Follow-up studies should be powered to examine important clinical outcomes.

Other limitations of our study include the fact that the measured fecal SCFA levels reflect a balance between SCFA production and absorption; the exact contributions of each cannot be determined from our measurements. Our study monitored SCFA for up to 30 days, at which time some SCFA were still reduced as compared to the baseline in the control group. We cannot determine how long it would take for the SCFA levels to return to the baseline, without monitoring longer time points. Additionally, SCFA composition has been shown to vary with dietary composition [49]. This study was not controlled for diet; however, diet information that was collected during the run-in period appeared to be similar among both the groups, and the participants were randomized. Furthermore, the microbiota analyses that are presented in this study, are relative rather than absolute measures. Thus, some of our results may be attributable to changes in the absolute, rather than the relative, abundance of the various members of the stool microbiota, as well as the total population size and stool biomass produced.

Our study demonstrated that both SCFA levels and microbiome changes were attenuated by the BB-12 yogurt. Importantly, *B. animalis* was not detected in the feces of subjects in the BB-12 group on days 21 and 30, although SCFA and microbiome changes persisted to day 30, which was the last day we examined. This suggests that BB-12 may have initiated changes to the microbiome, perhaps via the cross-feeding of resident microbes or other mechanisms, resulting in lasting, beneficial effects that were not dependent on high levels of BB-12. Future research should extend these findings, and include a more detailed metagenomic and metatranscriptomic analysis of the microbiota, while also expanding the clinical outcomes. Additionally, clinical applications of this research would be aided if future studies tested the timing of probiotic consumption during antibiotic use, since it is clear that BB-12 helps mitigate antibiotic disturbances.

## Figures and Tables

**Table 1:** Participant characteristics by group assignment.

Group		Control *n* = 20	Active/BB-12 *n* = 42	Not Randomized *n* = 4	Total *n* = 66
Age	Mean (sd)	29.4 (8.1)	29.6 (10.3)	31.8 (21.6)	29.7 (10.6)
Race	American Indian or Alaska Native	0	0	0	0
	Asian	3	6	3	12
	Native Hawaiian or other Pacific Islander	0	0	0	0
	Black or African American	5	8	0	13
	White	11	28	1	40
	More than one race	1	0	0	1
	Unknown/Not Reported	0	0	0	0
Ethnicity (Hispanic or Latino origin)	Yes	2	2	0	4
	No	18	36	4	58
	Unknown/Not Reported	0	4	0	4
Health insurance status	Yes	20	41	4	65
	No	0	1	0	1
Household smoking	Yes	0	2	0	2
	No	20	44	0	64
Marital status	Married	4	10	0	14
	Living with a partner	2	5	0	7
	Single	14	26	3	43
	Separated	0	0	0	0
	Divorced	0	0	0	0
	Widowed	0	1	1	2
Annual gross total income	Less than $15,000	1	6	0	7
	$15,000–$29,999	1	3	0	4
	$30,000–$49,999	1	5	0	6
	$50,000–$74,999	1	5	0	6
	$75,000–$99,999	5	6	0	11
	$100,000–$150,999	2	2	0	4
	$150,000–$200,000	1	1	0	2
	More than $200,000	2	6	3	11
	Prefer not to answer	0	3	0	3
	Unknown/Not Reported	6	5	1	12

**Table 2:** Fecal SCFA after antibiotics with and without BB-12.

	Control					Active/BB-12				
	Acetate (µM)					Acetate (µM)				
	Mean ± SD	Median	Range	*N*	% Change	Mean ± SD	Median	Range	*N*	% Change
Day 0	53.1 ± 18.0	53.0	22.1–85.9	12	N/A	45.2 ± 20.3	38.7	13.7–93.6	29	N/A
Day 7	42.3 ± 17.5	40.3	18.8–72.1	12	−20.3%	38.1 ± 17.6	39.6	4.4–74.8	27	−15.6%
Day 14	36.8 ± 11.1	33.6	23.9–60.5	12	−30.6%	40.8 ± 16.1	40.1	11.0–79.1	29	−9.7%
Day 21	43.8 ± 17.6	43.9	15.8–69.2	12	−17.6%	50.5 ± 20.9	50.9	18.8–98.9	29	11.7%
Day 30	39.8 ± 14.3	40.9	15.4–58.1	10	−25.1%	44.5 ± 14.1	47.2	18.1–66.1	26	−1.6%
	**Control**					**Active/BB-12**				
	**Propionate (µM)**					**Propionate (µM)**				
	**Mean ± SD**	**Median**	**Range**	***N***	**% Change**	**Mean ± SD**	**Median**	**Range**	***N***	**% Change**
Day 0	14.3 ± 9.1	12.2	3.8–34.0	12	N/A	13.0 ± 7.2	10.6	3.8–32.2	29	N/A
Day 7	14.3 ± 6.7	13.0	3.9–29.2	12	−0.3%	12.5 ± 6.0	12.5	<0.5–25.9	25	−3.8%
Day 14	11.8 ± 6.9	10.6	5.1–32.4	12	−17.3%	12.0 ± 6.1	11.0	3.7–31.8	29	−7.7%
Day 21	11.0 ± 3.5	11.2	5.7–18.9	11	−23.2%	14.8 ± 7.8	13.4	5.6–45.6	29	14.5%
Day 30	11.4 ± 7.3	8.4	4.5–27.3	11	−20.1%	11.5 ± 5.7	10.8	2.1–26.0	26	−11.6%
	**Control**					**Active/BB-12**				
	**Butyrate (µM)**					**Butyrate (µM)**				
	**Mean ± SD**	**Median**	**Range**	***N***	**% Change**	**Mean ± SD**	**Median**	**Range**	***N***	**% Change**
Day 0	12.7 ± 6.2	13.3	<0.5–21.0	11	N/A	12.7 ± 8.4	9.6	5.3–38.1	29	N/A
Day 7	9.0 ± 5.3	8.9	<0.5–16.4	11	−35.1%	8.3 ± 5.7	6.6	ND–22.6	24	−34.6%
Day 14	7.4 ± 4.3	5.9	2.2–16.8	12	−46.2%	8.9 ± 6.1	8.1	1.1–31.0	29	−29.7%
Day 21	11.6 ± 9.2	8.9	1.1–30.5	11	−16.4%	11.9 ± 7.8	10.8	1.1–37.1	29	−5.8%
Day 30	8.3 ± 5.3	6.3	0.8–19.0	11	−39.8%	9.9 ± 4.0	9.8	1.0–17.5	26	−21.6%

**Table 3:** Clinical symptoms by time point.

Time Point	Baseline Health			Post Run-in			Day 7			Day 14			Day 30		
Symptom, Number of Reports (%)	C ^†^	A ^‡^	Total ^§^	C	A	Total	C	A	Total	C	A	Total	C	A	Total
Group *n*	20	42	66	20	40	61	18	38	56	17	38	55	16	36	52
Loose stool	2	2	4	1	2	3	8 (44)	10 (26)	18	5 (29)	6 (16)	11	2	1	3
Diarrhea								1	1	1		1			
Constipation		3	3	1		1	2	2	4	2	2	4	1	2	3
Fever															
Flatulence	3	6	9		1	1	8	4	12	1	3	4	1	1	2
Lack/Loss of Appetite		3	3				2	1	3	1	3	4	1		1
Stomach Pain		1	1				3	1	4	1	1	2	1		1
Rash								1	1					1	1
Vomiting								1	1		1	1		1	1
Allergic Reaction		1	1												
Dyspepsia		1	1				4	1	5						
Nausea		1	1				5	3	8						
Other															
Headache							2	1	3		2	2			
Light-headed											1	1			
Migraine														1	1
Passing undigested food							1		1						
Rectal pain								1	1						
Ringing in ears											1	1			
Runny nose														1	1
Subconjunctival hemorrhage														1	1
Unable to fall asleep after waking at night											1	1			
Yeast Infection							1	1	2					1	1

^†^ C; control group. ^‡^ A; active/BB-12 group. ^§^ Total *n*; group size (includes non-randomized group *n* = 4; not shown).

## Data Availability

Data and materials used in the analysis are available upon request from the corresponding authors for the purposes of reproducing or extending the analysis. Sequence reads from the 16S rRNA gene profiling are available at NCBI Sequence Read Archive under accession number PRJNA668752.

## Supplementary Materials

The following are available online at https://www.mdpi.com/article/10.3390/nu13082814/s1, Table S1: nutrient profile of the BB-12-supplemented yogurt; Figure S1: linear discriminant analysis effect size (LEfSe) results in the BB-12-supplemented group; Figure S2: LEfSe results in the control group; Figure S3: individual SCFA expressed as a percent of total SCFA.

## Abbreviations

AAD	antibiotic-associated diarrhea
ASV	amplicon sequence variants
AUC	area under curve
BB-12	Bifidobacterium animalis subsp. lactis BB-12
HEI-2015	healthy eating index–2015
IND	investigational new drug
LEfSe	linear discriminant analysis effect size
SCFA	short-chain fatty acid
