# Supplementary material for: Bifidobacterium animalis subsp. lactis BB-12 Protects against Antibiotic-Induced Functional and Compositional Changes in Human Fecal Microbiome
Source: Nutrients. 2021 Aug 17;13(8):2814. doi: 10.3390/nu13082814 (PMC8398419; doi:10.3390/nu13082814)
Supplement: Supplementary file 1 [file nutrients-13-02814-s001.zip › nutrients-1337255-supplementary.pdf]

Supplementary Materials

Table S1 Nutrient profile of the BB-12 supplemented yogurt.

**Nutrition Facts**			
Serving Size: 100g			
**Amount Per Serving**			
**Calories** 110		Calories from Fat 10	
			**% Daily Value***
**Total Fat** 1.5g			**2%**
Saturated Fat 1g			**5%**
Trans Fat 0g			
**Cholesterol** 5mg			**2%**
**Sodium** 55g			**2%**
**Total Carbohydrate** 23g			**8%**
Dietary Fiber 0g			**0%**
Sugars 19g			
**Protein** 3g			
Vitamin A 0%	●	Vitamin C 2%	
Calcium 10%	●	Iron 0%	
*Percent Daily Values are based on a 2,000 calorie diet. Your daily values may be higher or lower depending on your calorie needs:			
	Calories:	2,000	2,500
Total Fat	Less than	65g	80g
Sat Fat	Less than	20g	25g
Cholesterol	Less than	300mg	300mg
Sodium	Less than	2,400mg	2,400mg
Total Carbohydrate		300g	375g
Dietary Fiber		25g	30g
Calories per gram:			
Fat 9 ● Carbohydrate 4 ● Protein 4			

Figure S1 Linear discriminant analysis effect size (LEfSe) results in the BB-12 supplemented group. Microbiota changes focusing on relative abundances of bacterial taxa were compared using LEfSe between two time points. (A) Post run-in baseline samples from the BB-12 group were compared to pre run-in baseline samples. No differentially abundant features found. (B-E) Post run-in baseline samples for the BB-12 group were compared to samples from: (B) day 7, (C) day 14, (D) day 21, and (E) day 30.

A **No differentially abundant features found.**

B

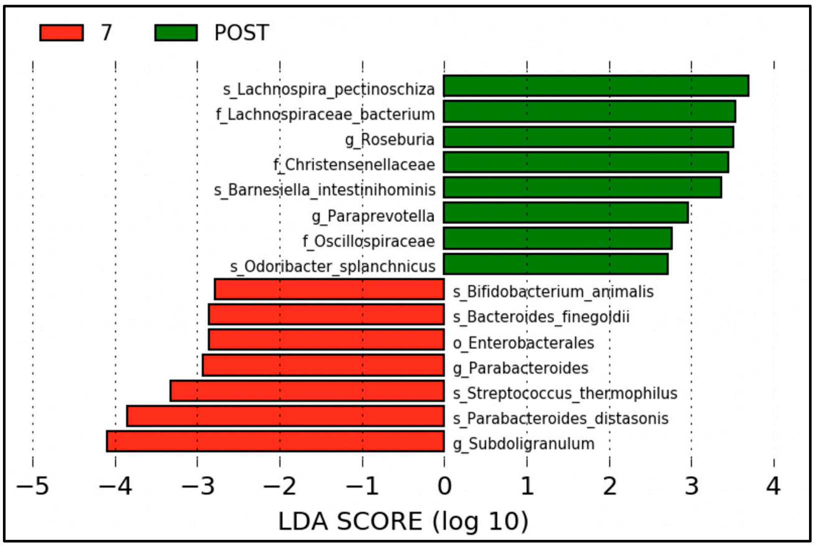

C

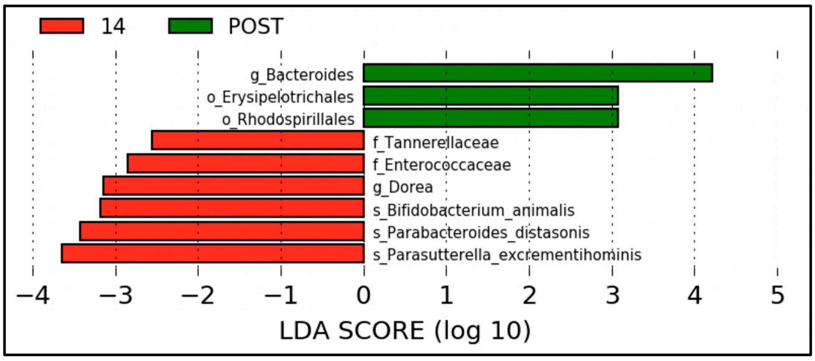

D

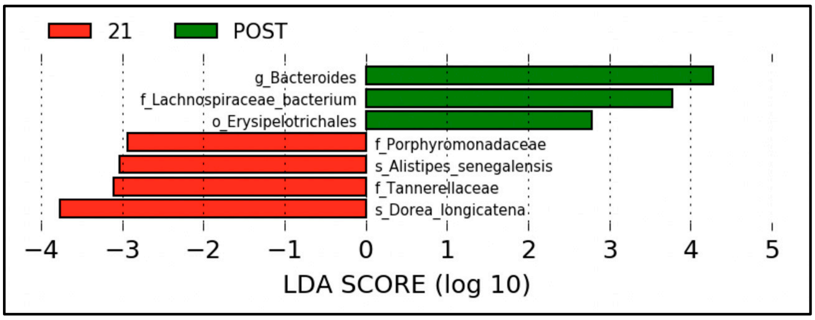
21
POST
g_Bacteroides
f_Lachnospiraceae_bacterium
o_Erysipelotrichales
f_Porphyromonadaceae
s_Alistipes_senegalensis
f_Tannerellaceae
s_Dorea_longicatena
−4
−3
−2
−1
0
1
2
3
4
5
LDA SCORE (log 10)

E

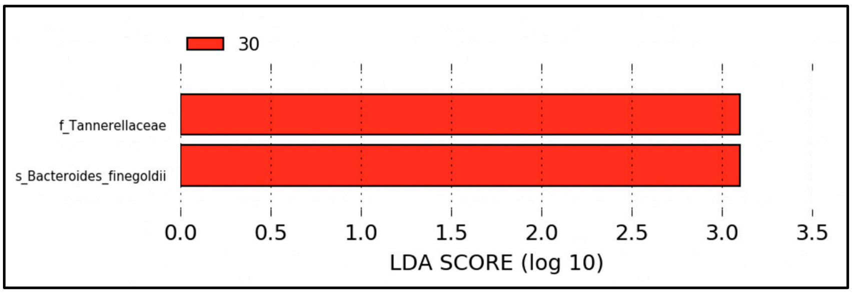
30
f_Tannerellaceae
s_Bacteroides_finegoldii
0.0
0.5
1.0
1.5
2.0
2.5
3.0
3.5
LDA SCORE (log 10)

Figure S2 LEfSe results in the control group. Microbiota changes focusing on relative abundances of bacterial taxa were compared using LEfSe between two time points. (A-E) Post run-in baseline samples from the control group were compared to samples from: (A) pre run-in baseline, (B) day 7, (C) day 14, (D) day 21, and (E) day 30.

A

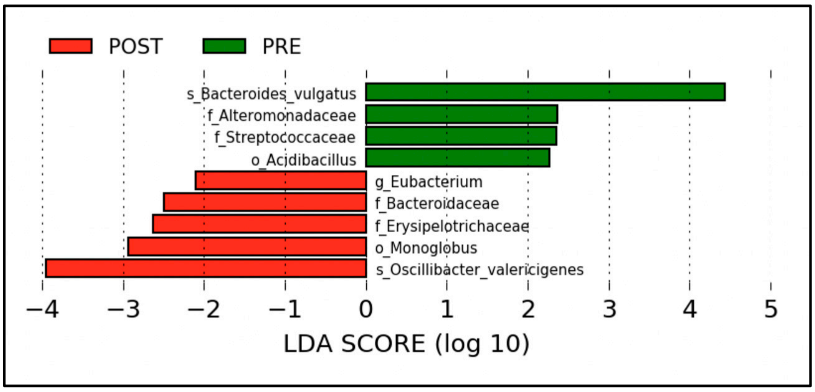

B

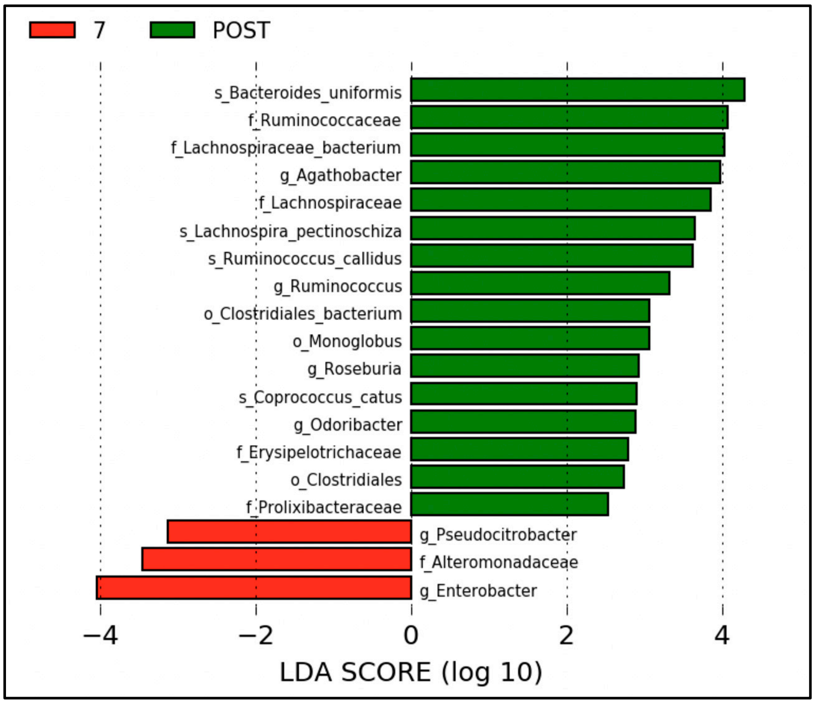

C

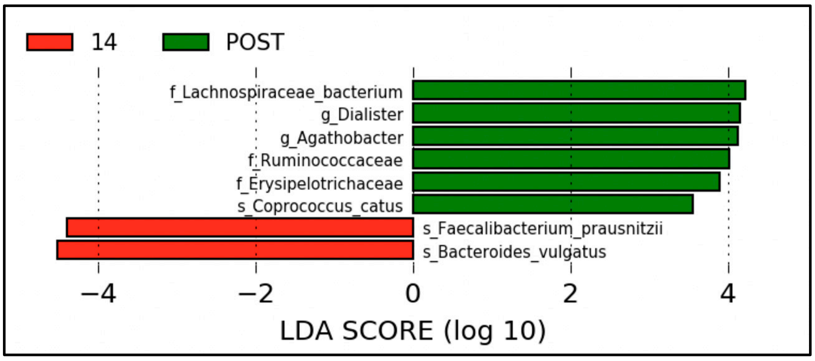
14
POST
f_Lachnospiraceae_bacterium
g_Dialister
g_Agathobacter
f_Ruminococcaceae
f_Erysipelotrichaceae
s_Coprococcus_catus
s_Faecalibacterium_prausnitzii
s_Bacteroides_vulgatus
−4
−2
0
2
4
LDA SCORE (log 10)

D

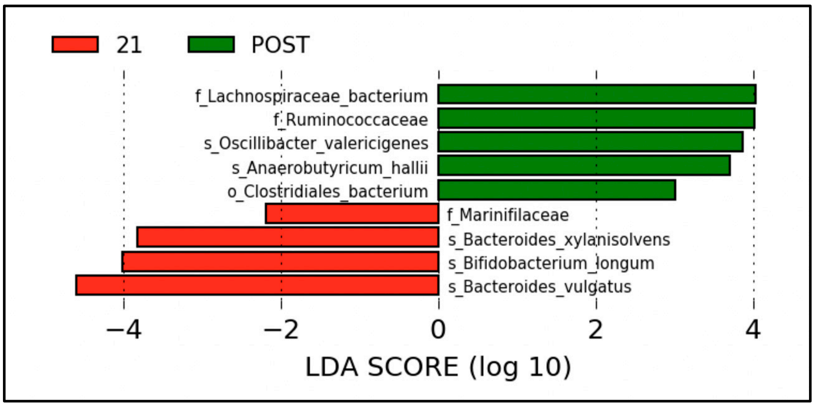
21
POST
f_Lachnospiraceae_bacterium
f_Ruminococcaceae
s_Oscillibacter_valericigenes
s_Anaerobutyricum_hallii
o_Clostridiales_bacterium
f_Marinifilaceae
s_Bacteroides_xylanisolvens
s_Bifidobacterium_longum
s_Bacteroides_vulgatus
−4
−2
0
2
4
LDA SCORE (log 10)

E

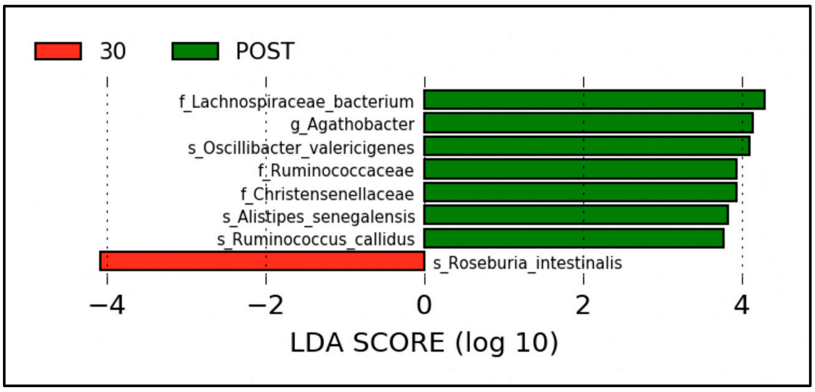
30
POST
f_Lachnospiraceae_bacterium
g_Agathobacter
s_Oscillibacter_valericigenes
f_Ruminococcaceae
f_Christensenellaceae
s_Alistipes_senegalensis
s_Ruminococcus_callidus
s_Roseburia_intestinalis
−4
−2
0
2
4
LDA SCORE (log 10)

Figure S3 Individual SCFA expressed as a percent of total SCFA. (A-B) Comparison of all SCFA over time with (A) Control, (B) Active. (C-E) Comparison of control and active for each individual SCFA with (C) acetate, (D) butyrate, (E) propionate. Total SCFA is the sum of acetate, butyrate, and propionate.

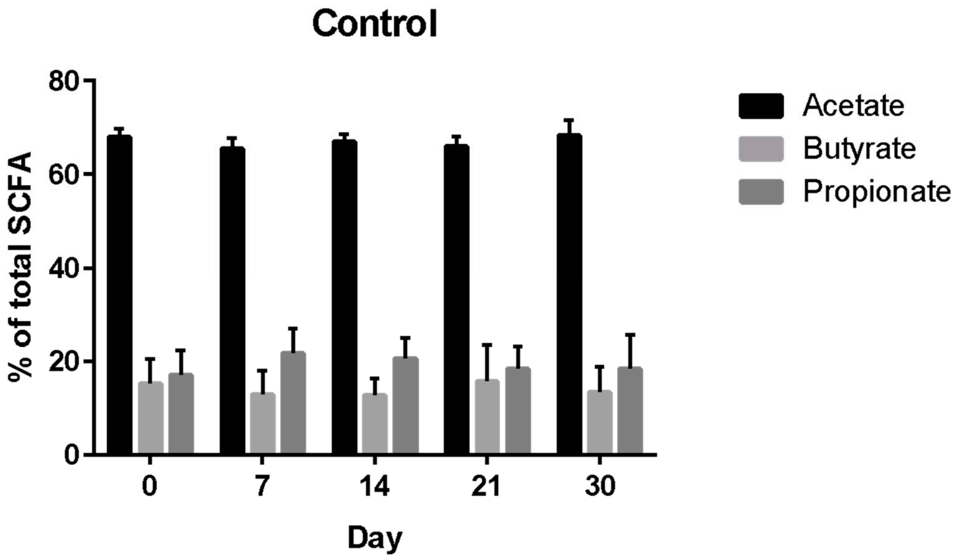

A

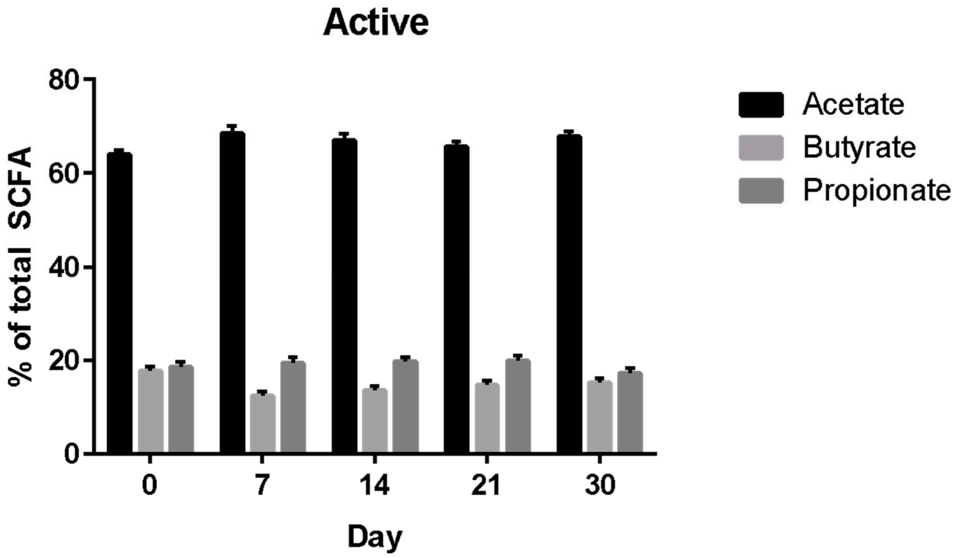

B

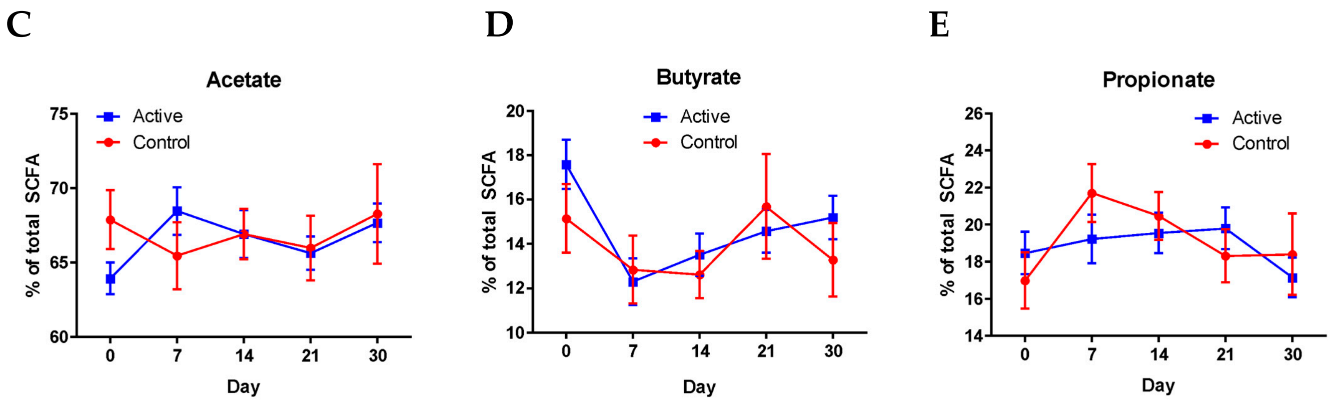
C
Acetate
Active
Control
% of total SCFA
75
70
65
60
0
7
14
21
30
Day
D
Butyrate
Active
Control
% of total SCFA
20
18
16
14
12
10
0
7
14
21
30
Day
E
Propionate
Active
Control
% of total SCFA
26
24
22
20
18
16
14
0
7
14
21
30
Day
